# Life threatening nontraumatic tension gastrothorax

**DOI:** 10.1002/ccr3.1468

**Published:** 2018-03-08

**Authors:** Ihab I. El Hajj, Marshall McCabe, DuyKhanh P. Ceppa, Jonathan A. Fridell, Stuart Sherman

**Affiliations:** ^1^ Division of Gastroenterology and Hepatology Indiana University School of Medicine Indianapolis Indiana; ^2^ Division of Cardiothoracic Surgery Indiana University School of Medicine Indianapolis Indiana; ^3^ Division of Transplant Surgery Indiana University School of Medicine Indianapolis Indiana

**Keywords:** Diaphragmatic rupture, esophagogastroduodenoscopy, gastrothorax, hiatal hernia

## Abstract

Tension gastrothorax is a rare condition, which poses a diagnostic dilemma and can be mistaken for a tension pneumothorax. Awareness of the risk factors, clinical presentation, and radiology findings of tension gastrothorax can help with the prompt identification and successful management of this life‐threatening condition.

## Case Description

**Figure 1:**
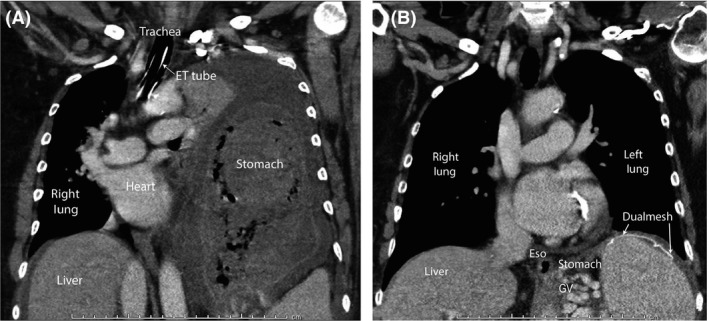
(A) Contrast CT chest image showing shift of mediastinal structures to the right, complete collapse of the left lung, near entirety of the stomach located within the left chest. (B) Contrast CT chest image postsurgical repair of diaphragmatic rupture with a Dualmesh and reduction of the hiatal hernia.

**Figure 2:**
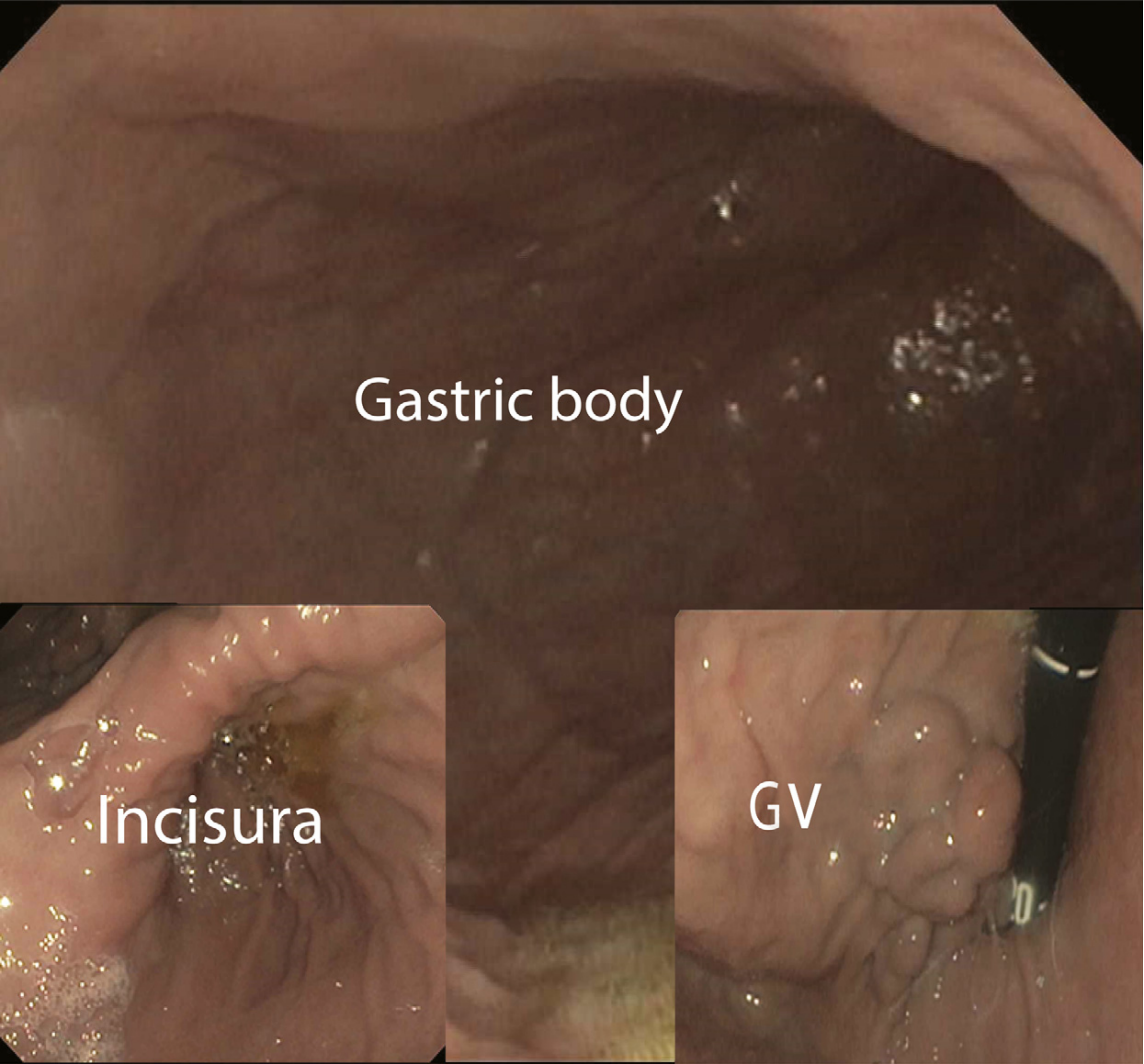
Endoscopy image of the stomach showing isolated gastric varices (IGV1), distended stomach with distorted anatomy.

A 66‐year‐old man with remote history of liver transplantation for nonalcoholic steatohepatitis cirrhosis, surgical repair of hiatal hernia (HH), and methylenetetrahydrofolate reductase (MTHFR) gene mutation on warfarin presented to the emergency department with acute onset chest and epigastric pain, shortness of breath, and melena. Vitals showed blood pressure 80/45 mmHg, pulse 120/min, respiratory rate 24/min, and oxygen saturation 89% on room air. The physical examination showed neck veins distension, heart sounds, and normal breath sounds heard to the right of the sternum, hyper‐resonant left hemithorax without breath sounds. Patient was intubated and resuscitated. A contrast CT of the chest and abdomen was performed. This showed a shift of mediastinal structures to the right, complete collapse of the left lung, near entirety of the stomach located within the left chest and confirmed the absence of pneumothorax or pulmonary emboli (Fig. 1A). Esophagogastroduodenoscopy (EGD) with limited air insufflation: gastric varices (GV) with white nipple sign and distended stomach with distorted anatomy (Fig. 2). Nasogastric tube was placed under endoscopic guidance and connected to suction. Patient underwent exploratory laparotomy. A large left‐sided diaphragmatic rupture was noted and repaired with a Dualmesh; the large HH was reduced. Dramatic clinical and radiographic (Fig. 1B) improvement was noted by the following day. The patient had a good recovery and remained symptom‐free for almost 18 months.

Tension gastrothorax is a very rare but potentially fatal clinical condition in which the stomach that herniates through a diaphragmatic defect into the thorax is massively distended by trapped air. It leads to severe symptoms due to the compression of the lung and mediastinum 1. Common etiologies include congenital diaphragmatic hernia occurring in childhood or adulthood and traumatic diaphragmatic injury (blunt or penetrating trauma) 2. Immediate clinical and radiographic evaluation should lead to accurate diagnosis followed by emergency decompression of the stomach before laparotomy with reduction in herniated viscera and repair of the diaphragmatic defect 3. An uneventful recovery can be expected postintervention.

## Authorship

IE, DC, JF, SS: involved in management of the patient, in revision of the manuscript, and approval of the final draft. MM: reviewed the literature and wrote the first draft.

## Conflict of Interest

None declared.
